# Sociability, Social Isolation, and Social Interaction During the First Months of COVID-19 Pandemic: a Qualitative Analysis of Brazilian, Finnish, and American Adults

**DOI:** 10.1007/s43076-022-00172-9

**Published:** 2022-03-23

**Authors:** Juliene Madureira Ferreira, Elisa A. Merçon-Vargas, Allegra J. Midgette

**Affiliations:** 1 Faculty of Education and Culture, Tampere University, Office 509, Åkerlundinkatu 5, 33014 Tampere, Finland; 2 Department of Human Development and Family Studies, University of North Carolina at Greensboro, Greensboro, NC USA; 3 Frank Porter Graham Child Development Institute, University of North Carolina at Chapel Hill, Chapel Hill, NC USA

**Keywords:** Virtual social interactions, COVID-19 pandemic, Sociability, Cultural values, Social isolation

## Abstract

The social distancing imposed by the pandemic transformed how people interact with others, and little is known about how it has impacted new ways of sociability and if culture influences this process. This is a qualitative study exploring changes in the configurations of social interactions and the resources for sociability that adults in Brazil, the USA, and Finland have developed during the initial stage of quarantine. A total of 95 participants (ages between 20 and 60) experiencing social isolation either living alone or with their partners (without children) completed online questionnaires about their interactive experiences. The questionnaire was composed of multiple choices, addressing the frequency, types, and length of social interactions before and during the pandemic, and open questions focusing on the participants’ experiences on online interactions during the pandemic. Frequencies were analyzed through a paired-sample *t*-test, and open-ended responses were thematically analyzed. Results revealed, first, that social isolation did not represent a significant change in the composition of the participants’ social network, but family bonds became the main connection during the period, and other sources of social interaction were kept due to the possibility of interaction through virtual means. Although the frequency of social interactions reduced, their significance increased. Second, virtual environments reframed social interactions, influencing individual’s bodily perceptions such as differences in attentional demands, communication processes, and awareness of their own image, and the interaction itself. Third, cultural values seemed to influence the way participants signified their interactive experiences. This study suggests that although virtual environments changed the ways interactions happen, virtual encounters were essential for maintaining participants’ social networks.

**Supplementary Information:**

The online version contains supplementary material available at 10.1007/s43076-022-00172-9.

## Introduction

The coronavirus pandemic (COVID-19) has greatly impacted human health and health systems, economic activity, and social life across the globe (e.g., Coibion et al., 2020; McDowell et al., 2020; Nyasulu & Pandya, 2020). In response to the pandemic, many countries imposed practices of social isolation and social distancing, which have been shown to potentially lead to negative mental health outcomes (e.g., Brooks et al., 2020; Wu, 2020; Xie et al., 2020; Zhou et al., 2020). With the rise and development of mobile technologies and easy access to the internet (e.g., via broadband), “being” online became more common (Nodari, et al., 2019). With the COVID-19 pandemic and demands for social distancing, online interactions became even more pervasive. The digital world we live in has several implications for information access, societal organization, and how we interact with one another. Despite the drastic changes in individuals’ day-to-day lives due to the pandemic, less is known about how individuals are adapting in a world where physical contact and bodily proximity are restricted and how they construct alternative ways to interact through digital tools.

In the present study, we investigated the social implications of the COVID-19 pandemic for single/living alone and married/cohabitating adults with no children in three different countries (Brazil, Finland, and the USA), with a focus on what their experiences reveal about changes in sociability based on virtual interactions. Although people have been encouraged to use digital tools as an alternative means for socializing, working, and even performing daily tasks (e.g., shopping, going to a museum, or working out), we do not know yet the consequences of virtual environments on sociability (the way people engage in social life). By investigating how individuals are experiencing social isolation, we can gain a better understanding of protective factors contributing to living in the new digital era under pandemic conditions. Such an investigation has implications also for how we understand the relevance of our bodily engagements in daily social interactions. By analyzing people’s social experiences in the absence of physical contact, it was possible to explore how they created new ways to establish engagement, shared understanding, and intimacy, which are important elements of human sociability.

### Social Experiences Through Digital Environments

Social experiences through digital environments are not new to the contemporary world. In fact, over the past decade, different studies have pointed to the increase in the use of digital tools in people’s daily lives, particularly for adolescents and young adults. For instance, a survey in the USA revealed that over 90% of teenagers owned smartphones and spent on average almost 7 h per day in front of screens (Jensen et al., 2019). They consume information, entertainment, and services; create and maintain social networks; and express their ideas, feelings, and engagements to the world through digital mobile tools, which constantly change the ways in which they can interact with others and with the surrounding world.

Several studies have pointed out the possible relationship between the excessive use of digital tools and unhealthy life habits (Kenney & Gortmaker, 2017; Nagata et al., 2020); problematic psychosocial behaviors, such as intolerance to uncertainty (Rozgonjuk et al., 2019); and symptoms of anxiety and depression, especially among younger populations (Elhai et al., 2019). Furthermore, a study examining the increasing use of virtual encounters suggested that the concrete physical distance during social interactions through digital tools can create psychological distance and disengagement, which influenced a person’s overall behavior during the interaction (Cartwright & Xue, 2020). Therefore, the extensive use of digital tools for social actions and interactions is a phenomenon that existed previously to the pandemic and has been argued to have a negative influence on human social behaviors, raising concerns among researchers and professionals in different fields.

Interestingly, however, during the COVID-19 pandemic, socializing through digital tools did not only increased but was encouraged as an alternative to maintain social interactions and perform social activities, such as working, shopping, and exercising (Riva et al., 2020). The use of technology was encouraged to alleviate the stress caused by compulsory social isolation (Goldschmitdt, 2020), prevent mental illnesses that are related to loneliness such as depression (Hawkley & Cacioppo, 2010; Killgore et al., 2020; Leigh-Hunt et al., 2017), and increase well-being (Gonçalves et al., 2020). In addition, technology has been used as a coping tool during the pandemic (Garfin, 2020).

Thus, further investigations are needed to explore how people transitioned (or are still transitioning) into different forms of sociability which now happen mainly through online applications, what are the issues they faced, and how they addressed them. During the pandemic, virtual encounters were, for many people, the only social connection possible, either due to the fear of self-contamination or in order to protect others (Petrocchi et al., 2021). This situation is very unique from different points of view, but particularly because digital applications change the physical environment where the interactions happen, positioning the bodies in a different space–time relation, limited by the features of the virtual setting. For that reason, exercising sociability mainly through virtual encounters mediated by digital tools may potentially change the way one sees the other and oneself in these interactions. This is especially so considering that cognitive processes are situated in socio-cultural institutions (e.g., legal, educational, and religious systems) that are supported by the procedures and social practices that comprise these institutions (Gallagher, 2013; Merritt et al., 2013).

### The Role of the Body During Social Interactions

The body plays an important role during social interactions. Emotions are expressed through the body and bodily movements, states of mind are shared, and one’s actions can be executed. The act of touching during social interactions, for example, is extensively used for different purposes such as controlling, comforting, and assisting others (Bergnehr & Cekaite, 2018); teaching culturally appropriate behaviors (Burdelski, 2010, 2020); or supporting and soothing people in stressful situations (Cekaite & Kvist Holm, 2017). Touch and body movements are used as an attention-getting device that enables people to construct participation frameworks in joint activities (Rautarinne et al., 2020). These and many other forms of bodily engagements are essential elements of human sociability, influencing the way we interact with others and serving as one of the most direct ways to define the boundaries between the self and the other (Kyselo, 2019).

Others’ bodily movement provides the insights necessary for guiding one’s behaviors and thoughts during social interactions. Interdisciplinary research has systematically demonstrated how our body shapes our perceptions, affording or constraining the experiences we entertain when relating to others (Uithol & Gallese, 2015), especially in situations of emotion recognition, joint attention, and action understanding (Reddy & Uithol, 2015). For example, adults coordinate in taking turns via non-verbal communication, evidencing the need for verbal synchronicity during dialogue (Brennan & Hanna, 2009; Shockley et al., 2003), and visually coordinate their attention through synchronized eye movements to understand each other’s actions when completing joint actions (Schneider & Pea, 2014). This coordination does not necessarily require any sophisticated skills, even when cognitive systems are involved. Action coordination is rather spontaneous, driven by the presence of others and even difficult to avoid in face-to-face joint activities. They can be present in different patterns of behaviors such as mirroring, anticipation, and imitation, which are related to the condition of the interaction rather than the individual’s decision to perform such a mechanism (De Jaegher & Di Paolo, 2008). Therefore, when comparing face-to-face to virtual interactions, the absence or the configuration of the bodily interaction in virtual encounters changes the possibilities of enactment (e.g., absence of eye gaze and touch), which defines different ways of perceiving the world and ourselves in it. This process is singular and unique and shows how human beings’ experiences are grounded by the specificity of the context in which they happen.

Furthermore, perception is not coterminous with the objective world but a form of interpretation, of meaning-making from what we experience, sense, and make sense, which is guided by what is important to us. Every individual navigates a sensory universe tied to personal history, to a culture and its specific ways to express feelings and intentions, a social context that includes rhythms of social life and extension of social networks, and a specific time and space (Breton, 2017). These elements build the references that define what is important for each individual during social encounters (Merritt et al., 2013), which will consequently influence how individuals interpret the many contextual changes imposed by the transition of face-to-face to virtual interactions (e.g., not being able to hug a friend in online meetings being interpreted as a sign of restriction, loss of intimacy, or absence of affection). That is so because, as stated by Kyselo and Tschacher (2014), “The world of humans is a world of others, so our social relations are what matter the most to us” (p. 2). For example, the use of cameras to mediate the visual stimuli during interactions is not merely a change in the way contact is made between people; it is a change in the way one perceives the others, behaves towards the others, and thus relates to oneself during the social interaction, and of course to the way in which others perceive and relate to that individual. Seeing loved ones through a camera during virtual interactions might evoke feelings of relief and excitement; they can use the resources of the camera to notice how one looks and comment on another’s appearance, state of mind, etc. But for colleagues at work, the change of interactions and use of video calls to hold business meetings would probably not matter much. In other words, what one feels about their social engagements depends on how people and others see themselves and others; it requires an implicit act of relationality (Moura et al., 2021). In this context, we are particularly interested in what and how the interactions through virtual environments affected one’s sociability.

### Different Contexts During the Pandemic: Finland, the USA, and Brazil

On 16 March 2020, the Finnish government declared a state of emergency and announced its first compulsory restriction to contain the spread of the coronavirus. Finland’s approach was designed upon the centralization of the guidelines for preventive actions. The restrictions consisted of a series of measures to decrease mobility within and throughout countries, including closing borders, closing of all schools from primary to higher education and commercial activities that were not considered essential for the population, imposed remote work for public and private sectors, and forbidden gatherings with more than ten people (Valtioneuvosto, 2020). Following this approach, great effort was made to flexibilize private and public services and make feasible the migration to digital platforms in all sectors; new work contracts allowing remote work and the use of digital tools to deliver services that demands interaction (e.g., expert consultancy, sales, teaching) were widely implemented. In this context, the required physical social isolation was followed with minor distress. The majority of Finns (73%) evaluated coping with the restrictions (social isolation and social distancing) fairly easily, and in comparison to other countries in the European Union, 84% of the Finns reported being satisfied with the measures adopted from March to July (Eurobarometer, 2020).

Both the Brazilian and US governments’ approach to addressing the pandemic markedly differed from that of the Finnish government. The strategy was originally based on decentralized decision-making, delegating responsibility to the states. In Brazil, on 3 February, the Ministry of Health released an ordinance declaring the COVID-19 pandemic as a public health emergency (Ministério da Saúde, 2020), but during the following months, the government regulated only public federal services (e.g., public education, health and justice system, and border control) and delegated to states the authority to decide on local policies. In the USA, the federal government imposed a national plan to control the pandemic, but the states held autonomy to decide on their regulations and strategies. In both countries, the restrictions varied considerably across the different states. In some cases, there were social isolation measures, which included closing commercial activities not essential to the population (e.g., bars, restaurants, and museums), and applying stricter mobility control. In other areas of the countries, social distancing and wearing masks in public spaces were the main safety measures; private markets and services remained open, and it was up to each individual to decide on self-protective behaviors. Private companies of all sectors, including schools, created their working regimes and adopted different strategies when implementing remote work.

Therefore, the three countries adopted different approaches for implementing social distancing and protection policies, as well as how to utilize digital tools to substitute for their social interactions. This means that the way people experienced social interactions during the pandemic could be considerably different, even living in the same country, especially for people in Brazil and the USA. Additionally, beyond the different political approaches and social realities, it is important to consider that specific features of these countries’ cultures might affect people’s needs to maintain their social networks, and how they experience social isolation in general. Social interaction is a defining part of a society’s culture, and it creates the behavioral agreements and mechanisms through which people orient themselves concerning others (Markus & Kitayama, 2010). Cross-cultural studies have revealed that patterns of behaviors, thoughts, and feelings are highly connected to culture and how the society continuously constitutes itself (Markus & Kitayama, 2010; Henrick, 2015). For example, Europeans are less likely than North Americans to associate happiness with personal achievement (Kitayama, et al., 2009), and seem to experience loneliness less intensively (Rokach & Neto, 2000). Therefore, although our focus is on understanding qualitatively the impact of social isolation on individuals’ sociality and not on establishing direct comparisons among the three groups, it is important to investigate social interactions across multiple cultural contexts during an unprecedented global event such as the pandemic.

### The Present Study

The present qualitative study aimed to investigate how the social isolation imposed by COVID-19 pandemic impacted people’s perceptions of their experiences in social interactions during the first months of the confinement. Our framework for the study considers two important arguments. First, the restriction to physically access social environments diminishes the affordances for social interactions and joint activities that are essential for establishment and maintenance of social bonds (Gaver, 1996; Merritt et al., 2013), which could impact in different changes in the configuration of peoples’ networks following the implementation of social distancing (Tibbetts et al., 2021). Physical distance has been the most frequently cited turning point negatively influencing long-distance friendships (Johnson et al., 2003). The dissatisfaction of not being able to have face-to-face interactions, and consequently the limitations to sustain self-disclosure, reciprocity, and mutual support, is one of the difficulties of maintaining long-distance social relationships (Vogt et al., 2005). Based on this understanding, we hypothesized that the relational bonds emerging from, or maintained by, physical affordances and social routines performed within specific spaces (e.g., professional relations in work environment, or friendships that are constituted by face-to-face interactions) would weaken with social isolation. Second, others’ bodily movement provides the insights necessary for guiding one’s behaviors and thoughts during social interactions, shaping our perceptions, affording or constraining the experiences we entertain when relating to others (Uithol & Gallese, 2015). Thus, interacting mainly online would change the way people perceive others during the interaction. We hypothesized that the lack of embodied engagements would be perceived as a hindering factor for communication processes and emotional engagement. Additionally, we also consider that culture influences how people see themselves in relation to social partners (Markus & Kitayama, 2010), and investigating qualitatively how individuals living in Brazil, the USA, and Finland will respond to the absence of physical contact and the use of virtual environments will provide important reflections how the pandemic changes cultural habits.

In an exploratory manner (Stebbins, 2001), this study investigated the initial transition between social life before and during the pandemic (retrospectively), focusing particularly on experiences marked by the first 3 months of the COVID-19 pandemic (April–June). The study addressed different aspects of the social interaction experience, such as the changes in social routines, perception of the use of virtual environments as an alternative space for social interaction, and self-report of participants’ feelings and thoughts during social isolation. The following research questions guided the present study:

- R1) How did physical social isolation and the use of digital platforms for social interactions influence the configuration of social networks during the first months of the pandemic?
- R2) How did physical distance and the use of digital platforms influence the way one perceived, acted, and enacted in interactions with others?

## Methodology

### Participants

A total of 95 individuals (average age 35.15; 82% female) from three countries, Brazil (*N* = 47), Finland (*N* = 25), and the USA (*N* = 23), participated in this study. Table 1 displays participants’ characteristics.

**Table 1:** Demographic information about participants

		Brazil (*n* = 47)	Finland (*n* = 25)	USA (*n* = 23)	Total (*n* = 95)
Age	Mean	36.09	31.84	36.83	35.15
	*SD*	10.68	6.33	9.78	9.61
Marital status	Married/cohabitating	28 (59.6%)	15 (60%)	16 (69.6%)	59 (62.1%)
	Single	16 (34%)	9 (36%)	7 (30.4%)	32 (33.7%)
	Missing data	3 (6.4%)	1 (4%)	0	4 (4.2%)
Gender	Female	36 (76.6%)	23 (92%)	23 (100%)	82 (86.3%)
	Male	11 (23.4%)	2 (8%)	0	13 (13.7%)
Housing location	City center	9 (19.1%)	6 (24%)	1 (4.3%)	16 (16.8%)
	Neighborhood close to city center	35 (74.5%)	11 (44%)	7 (30.4%)	53 (55.8%)
	Suburbs	3 (6.4%)	6 (24%)	13 (56.5%)	22 (23.2%)
	Rural area	0	2 (8%)	1 (4.3%)	3 (3.2%)
	Missing data	0	0	1 (4.3%)	1 (1.1%)
Educational level	Upper-secondary level (K-12)	3 (6.4%)	0	2 (8.7%)	5 (5.3%)
	Vocational training	6 (12.8%)	2 (8%)	1 (4.3%)	9 (9.5%)
	Bachelor’s degree	25 (53.2%)	5 (20%)	4 (17.4%)	34 (35.8%)
	Master’s degree	6 (12.8%)	14 (56%)	6 (26.1%)	26 (27.4%)
	Doctoral degree	7 (14.9%)	3 (12%)	10 (43.5%)	20 (21.1%)
	Missing data	0	1 (4%)	0	1 (1.1%)
Perceived social class	Poor	3 (6.4%)	1 (4%)	2 (8.7%)	6 (6.3%)
	Lower middle-income level	15 (31.9%)	4 (16%)	6 (26.1%)	25 (26.3%)
	Middle-income level	17 (36.2%)	18 (72%)	10 (43.5%)	45 (47.4%)
	Higher middle-income level	12 (25.5%)	2 (8%)	5 (21.7%)	19 (20%)
Working situation during pandemic	Employed, working from home	26 (55.3%)	18 (72%)	14 (60.9%)	58 (61.1%)
	Employed, working in the work environment	7 (14.9%)	3 (12%)	3 (13%)	13 (13.7%)
	Unemployed	4 (8.5%)	1 (4%)	3 (13%)	8 (8.4%)
	On unpaid leave	2 (4.3%)	0	0	2 (2.1%)
	On vacation	0	1 (4)	1 (4.3%)	2 (2.1%)
	Student	8 (17%)	2 (8%)	2 (8.7%)	12 (12.6%)
Work depends on in-person interactions	No	14 (29.8%)	11 (44%)	14 (60.9%)	39 (41/1%)
	Yes	32 (68.1%)	13 (52%)	9 (39.1%)	54 (56.8%)
	Missing data	1 (2.1%)	1 (4%)	0	2 (2.1%)
How often work dependent on in-person interaction	Sometimes	1 (2.1%)	1 (4%)	2 (8.7%)	4 (4.2%)
	Occasionally	7 (14.9%)	2 (8%)	1 (4.3%)	10 (10.5%)
	Every day	20 (42.6%)	8 (32%)	6 (26.1%)	34 (35.8%)
	Every week	4 (8.5%)	1 (4%)	0	5 (5.3%)
	Every month	0	1 (4%)	0	1 (1.1%)
	Missing data	15 (31.9%)	12 (48%)	14 (60.9%)	41 (43.2%)
Isolation type	Social distancing (advised to stay home)	43 (91.5%)	22 (88%)	19 (82.6%)	84 (88.4%)
	Horizontal isolation (imposed to stay home)	4 (8.5%)	3 (12%)	2 (8.7%)	9 (9.5%)
	Missing data	0	0	2 (8.7%)	2 (2.1%)
Days in quarantine	Mean	88.02	81.77	90.04	87.03
	*SD*	11.47	21.57	18.38	16.28

Our target sample was composed of adults (aged between 20 and 60) experiencing social isolation either living alone or with a partner (with no children), who had access to digital environments. Couples with children or households with multiple family members were excluded from the study as taking care of a child or others require different forms of social interactions (Castro et al., 2012), which adds complexity to the study that could not be addressed (e.g., children’s diverse needs for social interactions). Also, households with multiple family members impose the continuity of family bonds, impacting the analysis of the network configurations. We initially recruited 236 respondents, but after screening for complete answers and participants’ locations, 141 were excluded. The survey reached out to participants in 16 different countries, ten in the European Union (Austria, Belgium, Finland, France, Germany, Greece, Ireland, Lithuania, Spain, and Slovenia), four in the Americas (USA, Brazil, Mexico, and Canada), Thailand, and Australia.

### Procedures

Data was collected through an online survey (see Appendix A) managed through Qualtrics. Respondent-driven sampling (Heckathorn, 1997) was applied, and the initial convenience sample was created through the authors’ university email list, and by incenting respondents to share with their contacts from the target population to participate, resulting in a snowball effect. The link to the questionnaire was publicly available for 45 days (from May to June 2020) and was also distributed in different social media platforms (e.g., Facebook, Instagram). The survey was composed of 69 questions addressing demographic information and participants’ social interaction routines before and during the pandemic and open descriptive questions related to the interactive experiences during the pandemic. Social interaction routines were measured through two sets of questions addressing the frequency, extension, context, and the partners of social interactions before and during the pandemic. The questions were formulated allowing direct comparison of behaviors and social network information.

The open-ended questions addressed specifically participants’ perceptions of their sensations, feelings, and emotions during their virtual interactions; the differences between virtual and face-to-face encounters; the changes in the perception of others during the virtual interactions; and their perception of the importance of bodily contact during social interactions after experiencing being deprived of it. The open-ended questions were constructed specifically for this study grounded on the embodied cognition theoretical approach, which understands sociality as a process dependent on environmental embodied affordances (Gallagher, 2005; Gallagher & Lindgren, 2015); thus, the questions prompt participants to reflect and describe their perceptions of the experience of interacting (Appendix A). The survey was written in English and translated into Portuguese and Finnish. Translations were checked by native speakers in all languages. The survey was piloted in English and Portuguese with a small group of participants (age 23 > 45) from Finland, Brazil, and the USA. The pilot was used to improve the questions for clarity; thus, the pilot was not included in the dataset. The average time spent answering the questionnaire was 15 min.

### Analysis

**Fig. 1:**
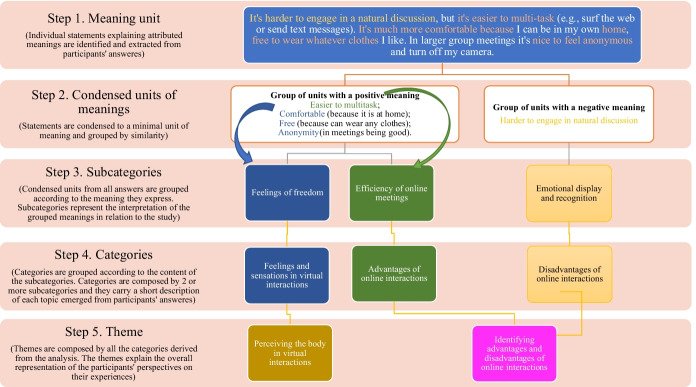
Example of the content analysis process

First, data analysis was conducted in SPSS to explore average frequencies of social interactions across countries. Paired-sample *t*-tests by country were conducted to investigate differences in types and frequency of interactions before and after the pandemic. In addition, we performed qualitative thematic analysis (Braun & Clarke, 2006) of the open-ended questions, which inductively (Hsieh & Shannon, 2005) reached to four central themes related to the main objectives of the study. For such analysis, participants’ answers were individually broken down into meaning units, carrying a singular content that can be interpreted within the context of the question. Different meaning units with similar contents were grouped forming condensed units of meaning, which were then interpreted within the context of the phenomenon under investigation generating a number of categories that explain the central theme (see Fig. 1).

### Coding and Reliability

The materials from the open-ended questions were translated to English by the first author and double-checked by a native Finnish speaker (for the Finnish dataset). Coding was carried out by the first author and checked (blind review) by an experienced research assistant. The coder agreement was 72.3% of 40% of the total data. Disagreements in code application were discussed, and a final consensus was reached. During open coding, each country’s dataset was treated separately for an in-group analysis and understanding of the phenomenon. The codes were merged in the axial coding process only if in-group analysis did not reveal discrepancy. Codes that did not belong to any theme in the in-group analysis were kept as a separate category until the end of the inter-group analysis, when it was inserted in the schema as a special subcategory of the most related theme. The axial coding then generated the map of categories explaining participants’ overall experiences with virtual social interactions (see Appendix B).

## Results

### Frequencies of Social Routines and Relational Bonds Before and During the Pandemic

Across countries, on average, participants reported engaging in social interactions through virtual environments and phone calls frequently (3–4 times a week; see Table 2 for description of social interaction types). Interactions in person outside or from home at a safe distance were not very common (rarely), which supports the idea that participants were indeed following social isolation recommendations. Participants reported to be engaging in social interactions, on average, somewhat less than before the pandemic. Moreover, participants on average believed that interactions during the pandemic were somewhat different from face-to-face interactions before the pandemic; they believed that their interactions during the pandemic were on average slightly more significant than before it, even though they considered the interactions during the pandemic as slightly less satisfying. From the viewpoint of embodiment and its notion that bodily engagements are essential features of sociality, virtual encounters during the pandemic could be perceived as less satisfying than the face-to-face ones before COVID-19. However, there are new forms of meaning-making, new value, or appreciation for virtual encounters as a result of the social restrictions and individuals’ limited opportunity to occupy different social spaces.

**Table 2:** Activity frequency before and after the pandemic (means and *SD*) by marital status across countries

Activity		Brazil	Finland	USA	Total
1.Communication with a co-worker(s) about work	Before	5.14 (1.64)	4.83 (1.95)	5.09 (1.51)	5.04 (1.68)
	After	3.98 (1.90)**	3.96 (1.90)**	4.13 (1.89)**	4.01 (1.88)**
	Cohen’s *d*	0.465	0.693	0.631	0.524
2.Meetings and other group tasks at work in person	Before	4.10 (2.00)	3.83 (2.08)	4.22 (1.78)	4.06 (1.95)
	After	2.71 (1.92)**	1.71 (2.20)**	1.65 (2.06)**	2.17 (2.07)**
	Cohen’s *d*	0.501	0.930	1.028	0.727
3.Chatting with a friend(s) outside the workplace	Before	4.10 (1.68)	4.21 (1.38)	4.22 (1.51)	4.16 (1.54)
	After	2.88 (1.94)**	3.67 (1.52)^†^	4.00 (1.65)	3.38 (1.81)**
	Cohen’s *d*	0.657	0.395	0.133	0.452
4.Social gatherings (with family or friends) in your house	Before	2.52 (1.25)	2.37 (1.26)	1.91 (1.44)	2.33 (1.40)
	After	1.00 (1.56)**	1.13 (1.70)**	0.35 (0.57)**	0.87 (1.44)**
	Cohen’s *d*	0.871	0.915	1.226	0.957
5.Social gatherings (with family or friends) in public establishments functioning during the day (e.g., cafeteria, library)	Before	2.48 (1.11)	2.88 (1.15)	2.43 (1.20)	2.57 (1.15)
	After	0.33 (0.82)**	0.71 (1.04)**	0.27 (0.55)**	0.42 (0.84)**
	Cohen’s *d*	1.369	1.645	1.987	1.560
6.Social gatherings (with family or friends) in public establishments functioning during the evening (e.g., bars, restaurants, nightclubs)	Before	2.52 (1.27)	2.21 (1.18)	2.68 (0.95)	2.48 (1.17)
	After	0.31 (0.75)**	0.33 (0.70)**	0.30 (0.64)**	0.31 (0.70) **
	Cohen’s *d*	1.275	1.348	2.355	1.457
7.Social gatherings (with family or friends) in public open spaces (e.g., parks)	Before	1.98 (1.17)	2.33 (1.20)	2.09 (1.19)	2.10 (1.18)
	After	0.40 (0.80)**	1.62 (1.25)*	1.35 (1.43)*	0.98 (1.23)**
	Cohen’s *d*	0.956	0.514	0.514	0.724
8.Social gatherings in other people’s houses	Before	2.31 (1.16)	2.12 (0.99)	2.00 (1.04)	2.18 (1.08)
	After	0.57 (1.02)**	0.65 (0.98)**	0.65 (0.83)**	0.61 (0.95)**
	Cohen’s *d*	1.104	1.022	1.067	1.073
9.Collective sports	Before	1.12 (1.60)	1.88 (1.70)	1.22 (1.68)	1.35 (1.66)
	After	0.31 (0.98)**	0.42 (0.93)**	0.26 (0.86)**	0.33 (0.93)**
	Cohen’s *d*	0.427	0.831	0.687	0.600
10.Time spent on social interactions (e.g., talking, messaging, engaging in activities) with all people	Before	1.53 (0.83)	1.38 (0.65)	1.43 (0.84)	1.47 (0.78)
	After	1.19 (1.03)**	1.33 (0.87)	1.30 (0.97)	1.26 (0.97)**
	Cohen’s *d*	0.447	0.105	0.172	0.293

Scores ranged from 0 = not part of my routine to 6 = several times a day

^**^p < 0.01; *p < 0.05; ^†^*p* < 0.10

Participants reported on average being slightly negatively impacted socially by the pandemic and by their limited ability to move freely. However, on average, participants also reported interacting with friends at about the same frequency as before the pandemic but interacting with the family slightly more during the pandemic, which supports the understanding that social networks were maintained although its configuration changed. Paired-sample *t*-tests revealed that participants in Brazil reported engaging more frequently before the pandemic than during it in the following activities: communicating with co-workers about work, participating in meetings and group tasks at work, chatting with friends outside the workplace, having social gatherings in their houses or others’ houses and in public places during the day and night, having a social gathering in public open spaces, and participating in collective sports (*p* < 0.05; effect sizes > 0.40). Similar results were found for participants in the USA and Finland (*p* < 0.05; effect sizes > 0.40), with the exception that, in these countries, participants reported chatting with friends outside the workplace at similar levels before and during the pandemic. Whereas participants in Brazil reported spending more time on social interactions before the pandemic, participants in Finland and in the USA reported engaging in social interactions at similar rates before and after the pandemic. Table 3 shows means for reported activities before and after the pandemic across countries.

**Table 3:** Quarantine impact on social interactions (means and *SD*) by marital status across countries

	Brazil	Finland	USA	Total
1.Frequency spent on social interactions during the quarantine through virtual environments (e.g., Zoom, Skype, WhatsApp); range from 0 = not part of my routine to 6 = several times a day	4.98 (1.14)	4.83 (1.47)	3.83 (1.53)	4.64 (1.41)
2.Frequency spent on social interactions during the quarantine through phone calls; range from 0 = not part of my routine to 6 = several times a day	4.21 (1.71)	4.00 (1.62)	3.65 (1.56)	4.01 (1.65)
3.Frequency spent on social interactions during the quarantine occasionally in person going outside; range from 0 = not part of my routine to 6 = several times a day	1.57 (1.38)	2.25 (1.19)	2.09 (1.56)	1.89 (1.40)
4.Frequency spent on social interactions during the quarantine from your home at a safe distance; range from 0 = not part of my routine to 6 = several times a day	1.12 (1.04)	1.17 (1.90)	2.04 (1.82)	1.37 (1.56)
5.Rate your interactions during the pandemic compared to before it; range from 0 = much less frequent to 6 = much more frequent	1.05 (0.94)	1.46 (1.32)	1.39 (1.44)	1.25 (1.19)
6.Significance of social interactions during the pandemic compared to before it; range from 0 = much less significant to 6 = much more significant	4.02 (1.19)	4.00 (1.10)	3.74 (1.10)	3.94 (1.14)
7.Social interaction satisfaction during the pandemic compared to before it; range from 0 = much less satisfying to 6 = much more satisfying	2.19 (1.24)	2.50 (1.29)	2.70 (1.33)	2.40 (1.23)
8.Feeling impacted socially by the pandemic; range from 0 = very negatively to 6 = very positively	1.60 (1.16)	2.04 (1.27)	1.96 (1.02)	1.81 (1.16)
9.Feeling impacted by restricted ability to move freely; range from 0 = very negatively to 6 = very positively	1.47 (0.96)	1.83 (1.24)	1.61 (1.16)	1.60 (1.09)
10.Frequency of interactions with friends comparing before and after the pandemic; range from 0 = much less to 6 = much more	2.44 (1.28)	3.25 (1.26)	3.00 (1.54)	2.80 (1.38)
11.Frequency of interactions with family comparing before and after the pandemic; range from 0 = much less to 6 = much more	3.26 (1.54)	3.88 (1.12)	3.26 (1.54)	3.42 (1.45)
12.Compare virtual interactions during the pandemic to face-to-face before the pandemic; range from 0 = not different at all to 3 = very different	1.91 (0.84)	2.00 (0.89)	1.96 (0.83)	1.94 (0.84)

To summarize, overall, social isolation did not have a great impact in the composition of the social network of our participants. Whereas family interactions became more central during the social isolation period, other sources of social interactions were maintained during the pandemic due to the possibility of interacting through virtual environments. Additionally, although the frequency of social interactions was reduced during the pandemic, their perceived significance increased.

### Qualitative Views on the Experience of Social Interaction in Virtual Meetings and Its Impacts on Social Encounters

The results from the qualitative analysis of open-ended questions showed that, among the 95 respondents from the three countries, only 2 (Finland 1, USA 1) openly stated not perceiving any differences between virtual and face-to-face interactions during social isolation, and not being affected at all by the social restrictions during the pandemic. The use of virtual environments did not affect the way they perceived others or the social interaction itself, and physical contact was not an important element in their social life.

**Fig. 2:**
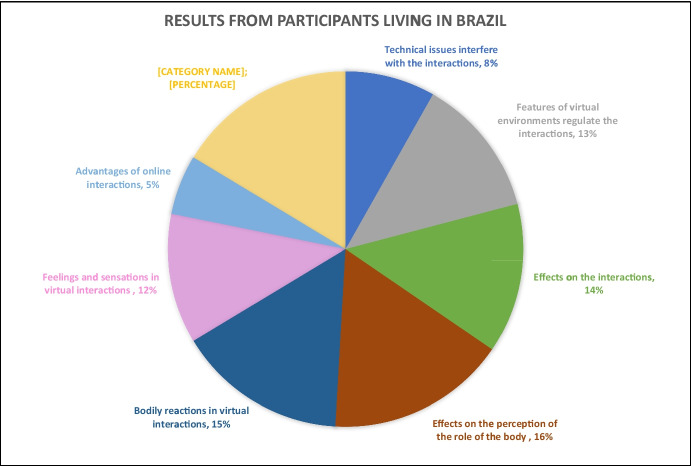
Results from participants living in Brazil

**Fig. 3:**
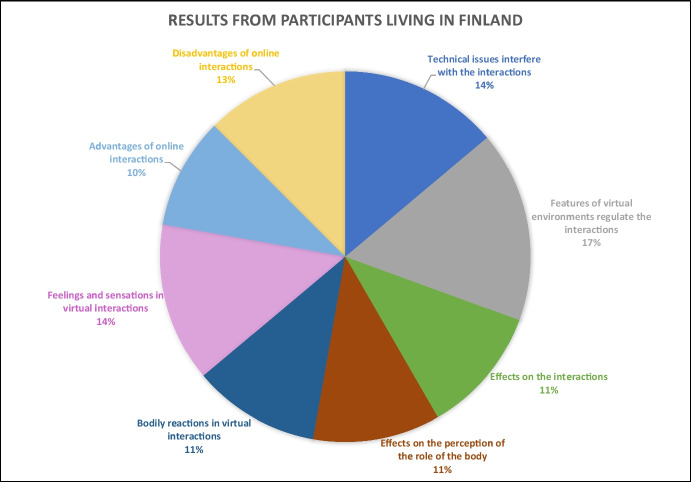
Results from participants living in Finland

**Fig. 4:**
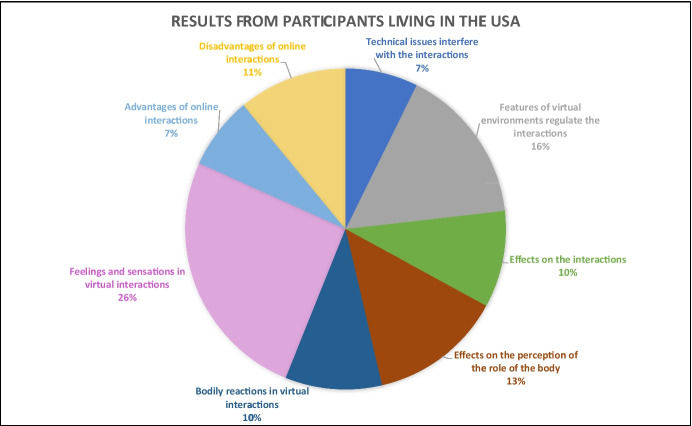
Results from participants living in the USA

For the remaining 93 respondents, the results portraying their experiences in online interactions (Figs. 2, 3, and 4) are examined through four central themes inductively elaborated from the open-ended questions of our survey: (a) *virtual* *environments reframing social interaction*, addressing participants’ views on how the virtual environment itself can impact interactions; (b) *effects of being socially isolated*, showing participants’ understanding of how being socially isolated impacts social interactions when they happen; (c) *perceiving one’s own body in virtual interactions*, addressing the sensations, feelings, and emotions evoked during virtual interactions; and (d) identifying *advantages and disadvantages of virtual interactions*, explaining participants’ perceptions of the differences between face-to-face and virtual interactions. We discuss each of these themes next (for the detailed content analysis scheme, see Appendix B).

#### Virtual Environments Reframing Social Interaction

Participants noted how *technical* *issues interfered with the interactions* and *how the features of the virtual environment itself regulated their interactions.* Both categories reveal participants’ views on the virtual environment as a setting that can impose limitations on their social encounters. The access to technology, as well as the knowledge on how to use, it is still limited, and the virtual environments, although not new to the participants, challenged the ways interactions were usually carried out, demanding a new set of interactive skills. The features of the virtual environments itself (e.g., visual stimuli transmitted through a camera, conversation dynamic defined by algorithms in the virtual platform) regulated participants’ ability to perceive stimuli, influenced participants’ self-awareness and self-consciousness, and prompted behavioral changes.

##### Constraints Related to the Technology and Constraints Related to Interactive Skills in Using the Technology.

These two different but complementary views described how participants from the three countries recognized their own (and others’) limitations on the usage of the tools for online encounters, especially in their personal life. Not all family members mastered the use of digital tools, especially older generations that had not developed the skills and knowledge to deal with communication technology. Reflected by the fact that the majority of our sample was middle to high middle class, access to digital tools was not an issue they personally faced in their network, but rather the emphasis was on accessibility to good internet providers. In particular, participants noted that momentary flaws (e.g., slow connections, low-quality video) due to internet connection affected the ways they could engage in dialogue and express their ideas. For example, Finnish participants noted that “[…] technology impact the flow of conversations because of delays, connection problems, video quality and so forth” (female, 26 years old, living alone) and that “bad connections made virtual interaction frustrating. As a teacher, teaching virtually was difficult since students were shy to interact and I was talking alone a lot without seeing their facial expressions (their cameras were often off)” (female, 29 years old, living alone). These are clear examples of technological limitations even among young adults with higher education and in a situation that allows access to internet services.

##### Perceived Stimuli

Interacting with others and the environment through a screen and mediated by a virtual platform that controls the presentation of visual and audio stimuli influenced the way participants perceived each other and the interaction. Most of the platforms used for online meetings show the video or picture of the speaker automatically, selecting the stimuli for the other participants or highlighting its features. This function, although meant to avoid overlapping sounds, imposes an attention pit and creates one shared focus for all members of the group; this is a situation that is uncommon in face-to-face interactions, where members of a group can take into account different references and gather a broader variety of stimuli. For the respondents across countries, this feature affected the interaction by narrowing down the type of information they could assemble during the meetings. For instance, a Brazilian participant (male, 33 years old, living alone) explained that “interactions by video conferences are limited, do not allow a broad experience, not only with the person but the surrounding environment; it makes plural interaction, with several people at the same time, impossible […].” It also disrupted the dynamics of the conversation. As illustrated by a Finnish participant (female, 36 years, cohabiting), “physical cues and reactions are less visible through virtual encounters and technical issues might disrupt the flow of conversation.” These statements above show the limitations of the interactive experience as a whole. Furthermore, losing control of selecting stimuli during the interaction affects its experience:

When we are interacting in person, several elements connect you to the person: the gestures, looking at where he is touching, objects present in the environment, etc. But talking to the person on video restricts all these other possibilities of interaction and it seems very tiring. The context where the person is, is not the same as yours. And vice versa. In a face-to-face meeting, the partners share a scenario, an environment, and this environment is incorporated into the interaction. (Brazil, female, 32 years old, cohabitating)

##### Self-awareness and Self-consciousness

Respondents also revealed that being on camera in a situation they know they will be heard and seen by everyone affects their self-awareness, particularly being more aware of or concerned about their physical appearances on camera and feeling shyer as a result. The participants from Brazil also expressed concerns related to the content of what they were saying, and how being on display during online meetings influenced their ability to express themselves. Self-awareness, in this context, is directly related to identifying one’s actions during the interactions, e.g., “[…] during virtual meetings I speak less; I’m quieter” (Brazil, female, 23 years old, cohabitating), and to the way to perform communication, e.g., “In virtual interactions, I believe that I am more concerned if people are understanding me correctly, so I think a lot more about how to express myself” (Brazil, female, 33 years old, living alone). Both processes are related to a set of bodily dependent communication patterns. Therefore, without the physical proximity that allows non-verbal cues into the interaction, being on camera (on display) increased their awareness of the content of the speech.

##### Behavioral Changes

When cameras were not being used, participants also claimed changes in how they perceived and acted during interactions. In this case, the absence of direct visual stimuli (e.g., seeing others’ faces and not sharing a common environment) affected their focus of attention. As stated by some participants, not having a visual reference “changes the way I behave because I know no one is seeing me. Makes me less present at the moment” (Finland, female, 38 years old, living alone), and “influence(s) the way I talk, as well as the content of communication” (Brazil, female, 22 years old, cohabitating). The lack of visual stimuli during the interaction prompted behavioral changes. These behavioral changes may be related to ensuring common understanding during the dialogue (e.g., not being able to see the other members of the group increases the behavior of checking if people are listening). It may also be related to self-regulation, as demonstrated in the following statements: “I also feel nervous about my ability to hear and be heard because my internet is not always stable, and I worry about missing information or not being heard” (USA, female, 30 years old, cohabitating); also,

Virtual encounters are different for me in terms of the ability to express myself physically, through gestures, mimics, etc. It has been especially challenging for me to talk to people who turned off their cameras during our conversations, as I like to see the reaction of the person I talk to. (Finland, female, 26 years old, cohabitating)

Despite the socio-cultural differences among the three countries, the participants’ views on how the virtual environments reframe social interactions highlights a change on who can be included in a conversation, how speakers feel during interactions, and how speakers perceive their behaviors.

#### The Effects of Being Socially Isolated

Participants revealed how social isolation had *effects on the perception of the role of the body* during the virtual interactions and *effects on the interaction* itself. Overall, the majority of the participants identified changes in their own perceptions and behaviors when interacting with other people online.

##### Noticing Their Own Body and the Absence of Other Bodies in the Interaction

Participants across countries perceived differently the absence of bodily contacts during social interactions such as hugs, sensory connection, hearing someone’s voice, and seeing someone in person. The majority of respondents living in the USA revealed that bodily contact “is not really important for most interactions” (female, 39 years old, cohabitating). For this group of participants, missing “making eye contact face-to-face” (female, 30 years old, cohabitating) and “being able to talk in person without fear” (female, 51 years old, cohabitating) seemed to be most relevant. Furthermore, respondents justified their answers stating that “I am not a(n) emotional person and am not one of those people that needs constant sensory connections to have a positive experience” (female, 32 years old, cohabitating) or “I’ve never really been a hugger, and I don’t particularly like to be touched except by people I’m close to” (female, 31 years old, living alone). These statements suggest that the need for physical contact may be an individual characteristic. For other participants in the USA, bodily contact “is extremely important for romantic partnerships” (female, 29 years old, cohabitating), and for interactions with family and friends; it “is what separates people I love from strangers” (female, 24 years old, cohabitating).

Among the Finnish respondents, physical contact was significant for the majority of the participants, particularly for encounters with friends and family. The need for physical contact was mainly justified as a way to prevent the feeling of being isolated and related to the feeling of calmness and relaxation. Examples of statements include “I noticed now, thinking about it, that physical contact calms me down” (female, 30 years old, living alone) and “having someone around makes me calm and relaxed” (female, 38 years old, living alone).

Participants from Brazil, on the other hand, heavily stressed the need for physical contact not just for individual engagement in daily interactions, but as an element related to a general human necessity. For this group of participants, social interactions can be defined as “what gives the sensation of concreteness” to the interaction (male, 33 years old, living alone). Physical contact is not only “part of communication” (female 29 years old, cohabitating) but also very important for people’s emotional state as “the deprivation of that contact has made people sad and anxious” (female, 31 years old, cohabitating). According to one of the Brazilian participants.

The absence of physical contact resulting from the quarantine generated an effective abstinence crisis. Whether by friends, the absence of dating, touching, hugging or even sexual partners. This abstinence of affection, in turn, leaves certain emotions more on edge and can cause certain emotional confusions, making you believe that your feelings are much deeper than they are in reality. I believe that before quarantine, it was easier to get a real sense of the meaning of our relationships and with social isolation, it gets a little bit confusing. (Male, 33 years old, living alone)

This finding is interesting considering that, overall, participants also reported an increase in the frequency of social interactions with family during the pandemic, when bodily contact was discouraged. In spite of being understood as an essential element for social interactions with close friends and family, the absence of bodily encounters did not prevent the social interactions to happen, or even to increase.

##### Emotional, Psychological, and Behavioral Changes

Participants in all three countries identified various changes in how they perceived themselves and others and their thoughts during social interactions through virtual environments. Such perceptions of change varied across and within the countries and targeted different aspects of the interaction; they did not indicate one unique direction or aspect in which change happened. In general, it either affected the way people experienced specific feelings while interacting, such as anxiety or distress, or influenced an overall state of mind that explains how participants are elaborating on this period of their lives, e.g., “I am in an abstinence crisis of social contact and my emotions are more accentuated” (Brazil, male, 33 years old, living alone).

Direct expressions of emotional changes, such as “I have more anxiety in all interactions now” (USA, female, 30 years old, cohabitating), were identified particularly among the participants in the USA, and were sometimes related to the pressures of looking well on camera and having their appearance judged online. For example, one participant noted, “I am more anxious about the way I appear in the eyes of others” (USA, female, 30 years old, cohabitating). However, it was also present in this group of participants a specific sentiment of relief coming from not having to deal with pitfalls of the working environments, e.g., “I actually felt pleased not having to navigate conversations in the workplace” (USA, female, 29 years old, cohabitating). Statements addressing this type of psychological and emotional change are also directly related to the category feelings and sensations in virtual interactions and were part of the larger theme *perceiving the body in virtual interactions*, and could be in parallel. Psychological changes were more evident in the statements from participants in Brazil and in Finland and are related to their personal performance during the online interaction. Interestingly, while in Brazil participants declared experiencing themselves as more restricted or limited, e.g., “being shyer to interact now” (Brazil, female, 42 years old, living alone), in Finland the opposite effect emerged. Finnish participants noted that the inhibitions one might encounter in face-to-face interactions were eased in the online setting. Participants noted behavioral changes included, “I think I am being more brave online” (Finland, female, 26 years old, cohabitating). Here we see two very different meaning-making processes in relation to the same event (adapting to the use — or not — of the cameras in virtual interactions). While for the Americans the camera was a source of anxiety and for Brazilians a source of fear, or perhaps embarrassment, for the Finnish participants, it prompted positive changes in their behaviors towards interacting with others in social encounters. We do not believe that these findings represent a universal norm for these social groups, and we recognize that personal trades or personal inclination for this meaning-making are the main driving force for these results. Nevertheless, it is important to remark that our bodily engagements are interpreted within a circumscribed social context. Individuals navigate a sensory universe tied to culture, which creates collective ways of responding to certain events or stimuli (Breton, 2017).

#### Perceiving One’s Own Body in Virtual Interactions

When questioned about the sensations, feelings, and emotions during virtual social interactions, participants described awareness of their *bodily reactions* and specific *feelings and sensations in virtual interactions* that helps us understand how changing the environment of the interaction from face-to-face to virtual impacted meaning-making of the interaction itself.

##### Signs of Discomfort and Attention Demands

The *signs of discomfort* were manifested by a large number of respondents across countries and included the description of mild sensations of tiredness, e.g., “It is boring and antsy” (USA, female, 39 years old, cohabitating) to signs of confusion and anxiety, e.g., “I am always eager to get off a video call even when I am excited to be on it” (Finland, female, 25 years old, cohabitating) or “[…] I can’t see people’s hands and how they are standing; staring at their faces throughout is unnatural for me” (USA, female, 30 years old, living alone). These signs of discomfort are reinforced by statements related to the different *attention demands* of virtual settings and how participants identified the need to be attentive to different signs of reciprocity during the interactions, e.g., “I need to pay much more attention to people’s features, the environment, and whether or not the person is paying attention to what I say. This all makes the virtual experience more tiring than the face-to-face” (Brazil, female, 32 years old, cohabitating). The increased attention demand and the feelings of discomfort could explain participants’ overall perception that virtual interactions are different, e.g., “The virtual interactions are very different. I don’t feel comfortable sharing my life virtually. Therefore, my conversations are shallow compared to how they were before” (Brazil, male, 27 years old, living alone).

In a different way, discomfort was also expressed together with the acknowledgement of absence of signs of intimacy particular to cultural contexts. For example, respondents from Finland mentioned missing that “just being silent together didn’t happen in online meetings” (Finland, female, 29 years old, living alone). We can understand this against the backdrop of the Finnish communication culture, in which silence is not only broadly accepted but, in many situations, required and expected. This participant’s statement calls attention to a number of culturally situated movements, habits, and traditions that are not being experienced during social isolation, but are important for the sense of belonging and engagement of a social interaction.

##### Feelings of Freedom and Connectiveness and Feelings of Discontentment and of Being Alone

In relation to the feelings and emotions that emerge during virtual social interactions, *feelings of freedom* were particularly described by people living in the USA; it entailed a positive notion of virtual interactions allowing people to be more relaxed and freer. This freedom came from the possibility of “wearing any type of clothes” (USA, female, 29 years old, cohabitating), including one “doesn’t need to wear a bra” (USA, female, 40 years old, living alone), and even experiencing the freedom of being anonymous in a meeting. This content did not appear in any of the Brazilian statements, and just one among the Finnish participants. Participants also expressed *feelings of connectiveness* when reflecting about their social experiences online. Although this feeling was not expressed extensively among Finnish and Brazilians, it showed the importance of being able to keep significant relational bonds and, in a way, even creating intimacy with people that were not close such as a co-worker. For example, “seeing other people in virtual encounters made me feel closer to my family” (Brazil, female, 31 years old, cohabitating), and “my relation with them is more intimate now because you can see part of other’s life (pets, kids, décor)” (USA, female, 31 years old, cohabitating).

In contrast, *feelings of discontentment* were particularly present among respondents living in Brazil. Brazilian respondents noted that virtual meetings were unpleasant because of the lack of spontaneity, fun, and affectionate connection; their statements emphasize the value of the personal contact, the multiple sensorial inputs necessary in the interaction, and the idea that without such bodily features the interaction is not real. For example, “I need to see and feel the other person near to me. The absence of physical contact is important to make everything real” (Brazil, male, 33 years old, living alone). Lastly, *feelings of being alone* were mentioned mainly by respondents living in Finland, and it explains how virtual meetings can create distance and lack of connectivity in the encounter. The lack of intimacy, the sensation of strangeness, and the feeling of less “love” portray the limitations of virtual meetings in creating closeness and the feeling of being together, e.g., “the quarantine made me feel like I lost something essential” (Finland, female, 32 years old, living alone).

The overall awareness of what we feel and how we perceive ourselves acting during social interactions determine how we respond to others and create meanings from the experiences we share (Merritt et al., 2013). Although the social bonds were maintained during social isolation, sociability was transformed. The new feelings and sensations emerging from the extensive hours of virtual interactions created new similar bodily demands but were signified differently between the social groups. This finding accentuates the need to understand the socio effects of social isolation deeply contextualized by culture and social norms.

#### Advantages and Disadvantages of Virtual Interactions

When answering how the virtual interactions differed from the face-to-face ones, participants perceived the use of virtual interactions in the context of social isolation. The results showed that *advantages* and *disadvantages* were equally stated across countries. Participants recognized both advantages and disadvantages in *accessibility* and *efficiency* of the virtual meetings. But a high number of respondents believed that *emotional display and recognition of others’ feelings* were the most significant limitations of this type of interaction.

Among the advantages was the perception that virtual meetings can bring people together, providing the opportunity to continue working from home and to maintain a frequent connection with family members. Additionally, interactions through virtual environments were seen as more efficient in work-related matters; respondents pointed out that for work, virtual interactions were more straightforward and the reduced visual stimuli during the interactions facilitated focusing on the task in hand, e.g., “work meetings are more focused and succinct” (USA, female, 30 years old, cohabitating).

Among the statements pointing out the disadvantages in virtual meetings, *accessibility and efficiency* were a concern to practical elements impacting the quality of the communication during the interactions. Participants noted the lower quality and length of the conversation, and the lesser fluidity and more truncated conversational dynamics. Respondents from Brazil stated that “it is more difficult to show objects, pictures, and yourself in virtual interactions” (female, 36 years old, cohabitating), and that “technology still disperses my attention, seeing my image reflected on skype, listening my own voice in WhatsApp messages, this type of things distracts me. I haven’t adapted to it yet” (male, 36 years old, cohabitating). Respondents also stated disadvantages related to *emotional display and recognition of others’ feelings*, particularly pointing out how it was difficult to express and understand others during virtual interactions. Statements such as “physical cues and reactions are less visible through virtual encounters” (Finland, female, 36 years old, cohabitating) and “it is more difficult to see the sincerity in virtual interactions” (Brazil, female 39 years old, cohabitating) portray the barriers for emotional display and recognition in virtual meetings. This provides evidence that specific behaviors considered important in face-to-face encounters were simply not possible to be identified, or not present in virtual ones. These limitations influence how participants perceived the interaction itself as a process that was less fun, less affectionate, and less intimate, e.g., “virtual interactions are less happy” (Brazil, female, 36 years old, cohabitating).

It is also important to consider that how we perceive our interactions with others is influenced by the socio-cultural frames we have previously discussed. The differences found in this study could be explained by specific social context. In Finland, the majority of our participants (72%) were working from home, which may increase the overall sense of stability that may affect their social interactions.

## Discussions

Social distancing is a new norm, at least until the majority of the world’s population is vaccinated against COVID-19. People who are able to maintain active routines and social bonds during the pandemic present less negative psychological effects and signs of anxiety (Dickerson, 2020), and are better able to follow social distancing measures. Thus, the use of digital technologies that allow social interactions and the performance of daily tasks was and still is very important when implementing social distancing measures. In this study, we investigated the social interaction experiences of single/living alone and married/cohabitating adults with no children in Brazil, Finland, and the USA during the first months of the pandemic. The study revealed that different forms of sociality based on virtual interactions supported the maintenance of specific social bonds but were limited in providing the experience of intersubjectivity of face-to-face interactions. We discuss our findings in relation to the research questions that guided this study.

How Did Physical Social Isolation and the Use of Digital Platforms for Social Interactions Influence Social Networks During the Pandemic?

This study shed light on how, across countries, the protocols of social distancing imposed as a measure to prevent the spread of the virus influenced the reconfiguration of individuals’ social routines and relational bonds. Differently from what was found in a previous work (Sikali, 2020), in the present study, the physical social isolation or social distancing did not prevent sociability; the use of digital tools and platforms allowed social interactions to continue happening, maintaining social networks. The physical social isolation experienced in this particular context in time influenced, however, the configuration of social networks and the frequency of social interactions. The most relevant findings were that the interactions with family members increased in all three countries, and that relational bonds such as those with co-workers or friends, which are sustained by the physical presence in specific spaces and joint activities, were maintained. Therefore, our hypothesis that social distancing would weaken specific relational bonds that are dependent on physical engagements and material affordances for joint activities was not confirmed during the timeframe of the study. Other studies carried out during the pandemic have reported conflicting findings related to the impacts of social distancing/isolation protocols in peoples’ sociality. Similar to our findings, Bond (2021) reports from a longitudinal study that social closeness was not affected by social distancing; participants who increased their mediated social engagement with friends also increased their social closeness during social distancing, maintaining social bonds. On the other hand, Kovacs et al. (2021) showed decreases in network density and global network size following this period of profound social isolation, and Pietromonaco and Overall (2021) argued that the decrease of social network density during social isolation protocols contributed to increasing harmful dyadic processes in stable relationships such as among couples. Elmer et al. (2020) suggested that specific worries related to COVID-19, isolation in social networks, lack of interaction and emotional support, and physical isolation are associated to negative mental health trajectories. The present study does not address health factors but contributes to the ongoing discussion of the impacts of social distancing protocols by showing how people experienced the social interactions online, revealing what were the challenges in maintaining their social bonds. Interaction depends on enactments that are defined by where, when, with whom, how, and why one interacts. This study reinforces the idea that when there are constraints on accessing in specific environments, or changes in one’s routine (e.g., migration of work to virtual platforms), the process of social participation changes.

Furthermore, the present study also opens room for discussing how culture, particularly distinct expectations for how to engage socially with others, may play an important role in defining the ways people construct meaning surrounding social distancing and virtual social experiences, particularly through the different meanings participants attributed to the embodied experiences during virtual interactions. Social networks are defined by the actions people perform in different social contexts they take part in, and they are organized and maintained within a specific socio-cultural frame (Rossetti-Ferreira et al., 2008). This shows the nuances that are very much related to a way of living and understanding what social interactions are and what they represent in one’s daily life. For instance, how each cultural community engaged in intimacy influenced their experiences of social isolation. Brazilian participants considered physical engagement as an essential part of communication and social life; without the embodied experience of closeness, participants felt disconnected and deprived from a real interaction. This group of participants also reported spending less time on social interactions during the pandemic. On the other hand, Finnish participants noticed the loss of shared silences during virtual interactions, the practice of which is an important component of Finnish interaction. As for participants in the USA, virtual encounters were also seen as an opportunity for more relaxed work interaction as people were able to see part of each other’s lives (e.g., their house, décor, or pets), showing the value of freedom and the appreciation for signs of connection outside the office environment. These examples show particularities of these groups’ cultural contexts and indicate how people interpret differently the challenges imposed by social isolation.

Previous cross-cultural studies have also pointed out the ways in which culture influences social interactions during the pandemic. For example, Dheer et al. (2020) found that culture and social structure influenced social behavioral responses to social restrictions; societies leaning towards collectivism, hierarchy, and restraint had greater success in implementing recommended behavior for protective measures, while those leaning towards individualism, autonomy, egalitarianism, and indulgence were less likely to follow recommended guidelines. Culture also plays an important role in defining social attitudes, which impacts engagement in social distancing measures (Jovančević & Milićević, 2020). In these prior studies, cultures were categorized following models that analyze specific dimensions of socio structures (Hofstede, 2011). In this study, the results do not support straightforward categorization of the countries (i.e., collectivist or individualist societies). Our findings revealed that participants’ experiences of social distancing and the effects on their routines and feelings involved more complex factors than suggested by initial cross-cultural studies that categorize countries into *individualist* and *collectivistic*. This study highlights that the expectations defined by cultural values influence or even shape the interactive experiences and because of that, they should be taken under consideration when we analyze the social impacts of the pandemic in different populations.

How Did Physical Distance and the Use of Digital Platforms Influence the Way One Perceived, Acted, and Enacted in Interactions with Others?

In relation to how the absence of physical interaction influenced the ways individuals performed (acted and enacted) virtual social encounters, this study confirmed what was expected—changing the configuration of the environment in which the social encounter takes place produced distinct sensation, feelings, and thoughts about others and the interaction itself. However, it was not the lack of physical contact per se, but the impossibility to enact the interaction through specific bodily engagements that influenced the participants the most. Participants noted, for example, that different from face-to-face interactions, in virtual meetings the conversations can be disrupted by technical matters and that a unidimensional flow of information defined its dynamic. Also, perceptions of time–space were unshared, and the absence of behavioral cues for joint actions (usually present in face-to-face interactions) and the increase of attention demands in virtual encounters generated discomfort.

We recognize that these findings are aligned with previous studies showing how the change from face-to-face to virtual environments can modify the behavioral cues of engagement and disengagement constructed through the rhythm of conversation (Auer, 1999). Such modifications are mentioned in studies that show that increases in tiredness and anxiety are related to the constant use of technology for social engagement on a daily basis (Drouin et al., 2020; Mheidly et al., 2020). Further, virtual environment itself can increase the attention demand and narrow the bodily exchange of information (Center for Scientific Review, 2020), suppressing the non-behavioral signs such as the prompt facial expression and feedback of others (Niedenthal et al., 2010; Osypiuk et al., 2018), which has been serving as a path of human’s communication since early development (Iverson & Goldin-Meadow, 2005). However, our findings connect one’s awareness of the lack of behavioral clues in online interactions to their emotional, psychological, and behavioral change during the interaction itself. It provides a glimpse on how individuals construct meaning of their intersubjective endeavors, a qualitative perspective that has been underemphasized in the context of the COVID-19 pandemic. Therefore, this study contributes to ongoing discussions on how virtual environments impose a distinct corporeal experience in the interactive process, which can affect not only the individual’s experience in the interactions but the different contexts in which the social encounters happen, creating new configuration of sociability for all parts involved in the process (Moura et al., 2021).

Moreover, this study’s findings highlight, first, the importance of further developing methodological approaches that incorporate individuals’ understanding of their social experiences in daily-life interactions into the investigation of the consequences of social isolation during COVID-19 pandemic, giving visibility to the different features of sociability that are culturally grounded. Second, it suggests that, beyond knowledge on how to use digital tools and information technology, a different set of skills, social awareness, and ways to engage with others has to be developed during virtual interactions. This has implications for interventions aiming to support individuals during the pandemic. Some strategies that can be used in interventions are creating training on communication skills adapted for the virtual setting and self-awareness, awareness of initial symptoms of fatigue and cognitive overload in long hours of virtual interaction, and ways to compensate for such demands to prevent overall burnout. Another strategy would be to develop digital tools that could better support the behavioral cues missing from the current virtual social interactions.

## Final Considerations

This study contributes to our understanding of individuals’ experiences in virtual environments and the use of such tools as an alternative for distant social interactions. Engaging in virtual encounters was important for the maintenance of participants’ social networks even if the configuration of this network discretely changed during the initial moments of the pandemic. Along with other studies (Goldschmitdt, 2020; Riva et al., 2020; Tesar, 2020), the present study suggests that digital platforms were widely used by individuals during the pandemic. The social restrictions during the pandemic exacerbated the situation in which digital environments substitute for collective physical spaces, enlarged the perspectives and ideas about virtual social interactions, and softened attitudes towards the extensive use of virtual tools in people’s daily routines. As the pandemic continues, and social practices are transformed due to this new reality, further studies should investigate these new ways of sociability and its consequences.

### Limitations

There are three important limitations to this study that should be considered. First, the use of virtual environments as an alternative for social interactions is contingent on the accessibility to technology and previous knowledge on the use of such resources. Therefore, considerations on the sample profile of the present study should be taken; the majority of people answering the survey were middle and upper middle class highly educated, executing jobs that allowed them to work from home. This combination of factors increases the possibilities of accessing online environments and the knowledge needed to use such tools, which supports alternative actions for social interaction during social isolation (Moura et al., 2021). For this sample, virtual interactions (i.e., video or voice calls) were a viable alternative for interacting both in professional and personal life, which does impact how individuals’ experience social isolation in general.

Second, the study’s scope is narrowed by its data collection timeframe (45 days) and circumstance (beginning of the pandemic). At the time of data collection, the transition to virtual environments had just started and many technical adjustments and perceptual learning has occurred since then. Furthermore, it is important to take into account that we asked participants to recall and report on their social interactions before the pandemic retroactively. Third, the sample size was small, varying significantly in the total number of participants per country. Moreover, the majority of our participants were female. Future research should consider employing more mixed methods with larger samples with a greater diversity of gender and SES backgrounds and in more countries. Moreover, more research is needed to see how social interactions may have changed as the pandemic continues and social distancing measures have been and continue to be implemented in various countries.

## Supplementary Information

Below is the link to the electronic supplementary material.

Supplementary file1 (DOCX 28 KB)

## Data Availability

The material is stored in the Finnish Social Sciences Archive.
