# Supplementary material for: Sociability, Social Isolation, and Social Interaction During the First Months of COVID-19 Pandemic: a Qualitative Analysis of Brazilian, Finnish, and American Adults
Source: Trends Psychol. 2022 Mar 23:1–33. Online ahead of print. doi: 10.1007/s43076-022-00172-9 (PMC8942390; doi:10.1007/s43076-022-00172-9)
Supplement: Supplementary file 1 — Supplementary file1 (DOCX 28 KB) [file 43076_2022_172_MOESM1_ESM.docx]

**Appendix A – Questionnaire**

**2. Frequency of social interaction**

The following questions are about comparing the social interactions in your routine **before** the pandemic with those **during the time of the Covid19 quarantine**. By interaction we mean the complete variety of situations where you have encountered, talked, and/or acted with another person for any period of time (e.g., ordering food by telephone, chatting with a friend, going to the movies with someone, doing multiplayer sports).

**Before the pandemic**

2.1 Please indicate how frequently the following situations happened:

(  ) several times a day,  (  ) once a day, (  ) 3-4 x per week, (  ) once a week, (  ) once a month, (  ) rarely, (  ) not part of my routine

a) Communicate with a co-worker(s) about work

b) Meetings and other group tasks at work in person

c) Chat with a friend(s) outside the workplace

d) Social gatherings (with family or friends) in your house

e) Social gatherings (with family or friends) in public establishments (e.g., cafeteria, shopping centers, library)

f) Social gatherings (with family or friends) in public open spaces (e.g., parks, open spaces for sports)

g) Social gatherings in other people’s houses

h)  Collective sports

i) other, please specify:______________________

2.2 Rank numerically (1 being most frequent and 7 being least frequent) the people with whom you interacted with **before the pandemic** (e.g., consider face-to-face encounters, calls, messages, and virtual meetings).

(  ) Family or partner

(  ) Friends

(  ) Co-workers and business partners

(  ) Service providers (e.g., teacher, physical instructor, doctor)

(  ) Significant partner (e.g., boyfriend, husband/wife/ life partner)

(  ) Neighbors and local community

(  ) Dates

2.3 How much time did you spend on social interactions (e.g., talking, messaging, engaging in activities) with other people? (mark just one)

(  ) Less than one hour per day

(  ) Between 2-4 hours per day

(  ) Between 5-8 hours per day

(  ) More than 8 hours per day

**During the quarantine**

2.4 Please indicate how frequently the following situations happened: (please, consider all interactions)

(  ) several times a day,  (  ) once a day, (  ) 3-4 times per week, (  ) once a week, (  ) once a month, (  ) rarely, (  ) not part of my routine

a) Communicate  with a co-worker(s)

b) Meetings and other group tasks at work

c) Chat with a friend(s) outside the workplace

d) Social gatherings (with family or friends) in your house

e) Social gatherings (with family or friends) in public establishments (e.g., cafeteria, shopping centers, library)

f) Social gatherings (with family or friends) in public open spaces (e.g., parks, open spaces for sports)

g) Social gatherings in other people’s houses

h)  Collective sports

i)  other, please specify:____________

2.5 Rank numerically (1 being more frequent and 7 being less frequent) the people with whom you interacted with the most **during the pandemic** (consider interactions by messages/calls and virtual meetings).

(  ) Family

(  ) Friends

(  ) Co-workers and business partners

(  ) Service providers (e.g., teacher, physical instructor, doctor)

(  ) Significant partner (e.g., boyfriend, husband/wife/ life partner)

(  ) Neighbors and local community

(  ) Dates

2.6 How much time do you spent in total with social interactions with other people in general? (mark just one)

(  ) Less than one hour per day

(  ) Between 2-4 hours per day

(  ) Between 5-8 hours per day

(  ) More than 8 hours per day

2.7 How often have you engaged in social interactions during the quarantine?

(  ) several times a day,  (  ) once a day, (  ) 3-4 times per week, (  ) once a week, (  ) once a month, (  ) rarely, (  ) not part of my routine

a) through virtual environments (e.g., Zoom, Skype, WhatsApp video)

b) through phone calls

c) occasionally meeting face-to-face when going outside

d) from my house with a safe distance (e.g., talking to the neighbors from the balcony

e) Other:_________

2.8 Have you been seeking more or less interaction with your friends than before the crisis? (consider also online meetings)

Much less / somewhat less / slightly less / the same / slightly more / somewhat more / much more

2.9 Have you been seeking more or less interaction with your family than before the crisis? (consider also online meetings)

Much less / somewhat less / slightly less / the same / slightly more / somewhat more / much more

2.10 Do you think your social interactions with others have been more or less significant to you than before the crisis?

Much less / somewhat less / slightly less / the same / slightly more / somewhat more / much more

2.11 Have your social interactions with others been more or less satisfying than before the crisis?

Much less / somewhat less / slightly less / the same / slightly more / somewhat more / much more

2.12 Have you been feeling positively or negatively impacted socially?

(  ) very negatively, (  ) somewhat negatively, (  ) slightly negatively, (  ) no change, (  ) slightly positively, (  ) somewhat positively, (  ) very positively

2.13 Have you been feeling positively or negatively impacted by your restricted ability to move freely?

(  ) very negatively, (  ) somewhat negatively, (  ) slightly negatively, (  ) no change, (  ) slightly positively, (  ) somewhat positively, (  ) very positively

2.14 How would you compare your virtual interactions (e.g., Zoom, Skype, or other) **during the quarantine** to your face-to-face interactions before the pandemic?

Very different / somewhat different / slightly different / not different at all

**3. Experience of interaction during the pandemic**

**Prompting statement:** Please take some time to think of the experiences you had/having in the weeks of the Covid19 pandemic. Think about how you engaged in conversations, how you connected (or not) to people, what made/makes the experience different than before the quarantine, and if there was/is anything you learned during these interactions. When you have identified these experiences, please think about it for another second, until you feel you remember it well.

3.1 What makes the virtual encounters (e.g., Zoom, Skype, or other) different from face-to-face interactions for you? Does it change the way you talk, behave, or the content of the conversation?

3.2 What sensations, feelings and emotions did you experience having to interact with others virtually or respecting social distancing? Have you noticed any difference on how you perceive other people during the virtual interactions?

3.3 How important do you think the bodily contact (e.g., hugs, sensory connections, hearing someone’s voice, seeing someone in person) are for your interactions considering the experiences you had during the quarantine? Why?

3.4 What did you do to feel connected to other people during the quarantine? Please describe the actions you took and why.

3.5 Did you notice any changes in your behavior (e.g., spoke to yourself, got attached to objects that made you feel better, started taking care of animals or plants) because of social isolation? Please describe the behaviours and why they were important for you?

3.6 Considering all the interactions you had, please describe one experience that was particularly significant for you, and if you feel comfortable answering: What was special about it?

3.7 What are you grateful for during this time? Why?

3.8 How has this changed your connection to other people and/or your sense of connection to the world? Why?

**Appendix B – Content analysis schema**

| **THEMES** | **CATEGORIES** | SUBCATEGORIES | Number of units of meaning | | | Example of units of meanings  **Red =USA, Green=Brazil, Blue=Finland** |
| --- | --- | --- | --- | --- | --- | --- |
|  |  |  | BRA | FIN | USA |  |
| **1. VIRTUAL ENVIRONMENTS REFRAMING SOCIAL INTERACTIONS** | **Technical issues interfere with the interactions** | Constrains related to technology | 4 | 8 | 4 | *“Technology impacts the flow of conversations because of delays”*  *“Technology is limited for some people”* |
|  |  | Constrains related to interactive skills | 5 | 2 | 2 | *“Older people can’t use the software correctly; they don’t know how ”* |
|  | **Features of virtual environments regulate the interactions** | Perceptions of stimuli | 7 | 4 | 5 | *“Not seeing others makes me less present in the situation”* |
|  |  | Self-awareness and self-consciousness | 2 | 4 | 6 | *“I am more concern with my appearance on camera”* |
|  |  | Behavioral changes | 5 | 4 | 2 | *“Virtual interactions make me more shy to talk”* |
| **2. EFFECTS OF BEING SOCIALLY ISOLATED** | **Effects on the interactions** | Emotional and psychological changes | 12 | 5 | 4 | *“The quarantine made me feel like I lost something integral”*  *“I am judging myself more”* |
|  |  | *Presence of the partner being important | 3 | 3 | 4 | *“I feel very lucky to have my husband to quarantine with”* |
|  | **Effects on the perception of the role of the body** | Important bodily elements for the interaction | 7 | 3 | 5 | *“Physical contact is part of communication”* |
|  |  | Identifying the need of physical contact during the quarantine | 11 | 3 | 5 | *“I have missed hugging my family and friends now”* |
|  |  | The need of physical contact depends on peoples’ personality | 0 | 2 | 1 | *“I am not one of those people that need sensory connection to have a positive experience”* |
| **3. PERCEIVING ONE’S OWN BODY IN VIRTUAL INTERACTIONS** | **Bodily reactions in virtual interactions** | Signs of discomfort | 8 | 4 | 2 | *“More tiring”* |
|  |  | Attention demands | 9 | 4 | 6 | *“My attention decreased during the interaction”* |
|  | **Feelings and sensations in virtual interactions** | Feelings of connectiveness | 4 | 1 | 7 | *“I can see more of their personal life”* |
|  |  | Feelings of freedom | 0 | 1 | 6 | *“Can interact from my house, wearing any type of clothes”* |
|  |  | Feelings of discontentment | 8 | 3 | 5 | *“There is no spontaneity (missing the life from the interactions)”* |
|  |  | Feelings of being alone | 1 | 5 | 1 | *“Virtual interactions doesn’t give me the feeling of being together”* |
| **4. IDENTIFYING ADVANTAGES AND DISADVANTAGES OF VIRTUAL INTERACTIONS** | **Advantages of online interactions** | Accessibility | 3 | 5 | 2 | *“Virtual environments (accessibility) bring people together”* |
|  |  | Efficiency | 3 | 2 | 4 | *“Virtual meetings can be more efficient since the focus is on the task at hand”* |
|  | **Disadvantages of online interactions** | Accessibility | 1 | 2 | 2 | *“It is more difficult to show objects and pictures during online interactions”* |
|  |  | Efficiency | 7 | 4 | 5 | *“Conversation content is of lower quality (due to the entire circumstance)”* |
|  |  | Emotional display and recognition | 10 | 3 | 2 | *“Physical cues and reactions are less visible through virtual encounters”* |

*The content of this subcategory derives exclusively from participants experiencing social isolation cohabiting without children.
